# Editorial: Personality disorders in youth: from early diagnosis to treatment

**DOI:** 10.3389/fpsyt.2025.1558277

**Published:** 2025-02-03

**Authors:** Tianhong Zhang, Katherine Durham, Paula Yanes-Lukin

**Affiliations:** ^1^ Shanghai Mental Health Center, Shanghai Jiaotong University School of Medicine, Shanghai Engineering Research Center of Intelligent Psychological Evaluation and Intervention, Shanghai Key Laboratory of Psychotic Disorders, Shanghai, China; ^2^ Department of Psychiatry, New York State Psychiatric Institute (NYSPI), New York, NY, United States

**Keywords:** personality, diagnosis, intervention, youth, adolescent

The critical underdiagnosis and undertreatment of personality disorders (PD) in youth, which affect approximately 12-14% of adolescents, is a pressing issue. Despite evidence supporting the effective diagnosis and treatment of these disorders, there remains a tradition of delaying identification until adulthood. Recent therapeutic adaptations, such as Dialectical Behavioral Therapy for Adolescents and Mentalization-Based Therapy, offer promising tools for clinicians. However, misconceptions about the ability to diagnose personality disorders in younger populations, concerns regarding stigma, and the tendency to attribute symptoms to normative adolescent development hinder clinical practice. This Research Topic aims to enhance the understanding of personality disorders in youth, promote research on PDs less studied relative to Borderline Personality Disorder (BPD), and provide practical guidelines for identification and treatment while reducing stigma.

This Research Topic includes one conceptual analysis and four original research articles that explore various aspects of personality disorder research in youth across different countries. Together, they provide valuable insights for future studies and offer practical recommendations to enhance efficacy and improve outcomes for young individuals.

## Conceptual analysis

Hutsebaut et al. describe the ethical imperative to diagnose and treat PD in adolescents, emphasizing that such diagnoses are not only scientifically valid but also morally justified. The authors present seven key arguments supporting early intervention, highlighting the significant impact of PD features, particularly those of BPD, on predicting current and future psychopathology. Often making comparisons to medical illnesses, they argue that addressing these disorders during adolescence can prevent long-term psychosocial and health problems, and advocate for a shift from a stepped-care approach to a staged-care model that better meets the complex needs of young individuals.

Furthermore, the authors address the common stigma associated with PD diagnoses, asserting that early detection and intervention may actually reduce stigma over time, as seen in other areas of healthcare. They contend that regular mental health services are often inadequate for young people with PD, leading to unmet needs and worsening outcomes. By promoting sensitivity in diagnosis and treatment, the paper aims to foster a culture that supports young people with personality disorders, ultimately contributing to more effective and just care for this vulnerable population.

## Original research

The original research by Gajwani et al. investigates the vulnerabilities of young people aged 15 to 25 who are at risk of serious mental illness, focusing on BPD and first episode psychosis. Conducted as a cross-sectional study within the United Kingdom’s mental health services of the National Health Service, the research reveals a high prevalence of adverse childhood experiences and emotional dysregulation among participants, with significant associations between these factors and neurodevelopmental disorders such as attention deficit hyperactivity disorder and autism spectrum disorder. Additionally, findings indicate that emotional regulation mediates the relationship between both adversity and BPD, as well as the association between neurodevelopmental disorders and BPD. The findings suggest that emotional dysregulation may serve as a critical early marker for future clinical severity in this population, highlighting the need for routine screening and early intervention to improve outcomes.

Recognizing the long-term consequences of adverse childhood experiences is crucial for understanding the vulnerabilities faced by young people. This is further explored in the research by Fellinger et al. who investigate the impact of adverse childhood experiences on young adult psychiatric inpatients, highlighting the increased risk factors associated with recurrent admissions. The study involved a systematic chart review of 390 psychiatric inpatients aged 18 to 25, revealing that those with previous child and adolescent psychiatric inpatient treatment had significantly higher rates of adverse childhood experiences, including family dysfunction, neglect, and abuse, particularly sexual abuse. The findings indicate that these individuals are more likely to suffer from comorbid mental health disorders and experience greater functional impairments, emphasizing the need for targeted interventions to address the effects of adverse childhood experiences in this vulnerable population.

The importance of effective assessment tools for BPD in adolescents is further highlighted by the research conducted by (Zhuo et al.). This study evaluates the validity and reliability of the Chinese version of the Borderline Personality Features Scale for Children-Short Form, involving 120 adolescents diagnosed with BPD. Through confirmatory factor analysis and various reliability tests, the researchers demonstrated that the scale is a valid and reliable instrument for assessing borderline personality features among Chinese adolescents, ultimately aiming to enhance early diagnosis and intervention strategies to improve clinical outcomes for this population.

Building on the growing interest in the relationship between childhood experiences and mental health outcomes, the study by Wang et al. investigates the prevalence and distinctions of PD and childhood maltreatment among adolescents and adults diagnosed with psychotic or non-psychotic disorders. It highlights significant differences in self-reported PD traits and experiences of childhood maltreatment between these age groups. The findings indicate that adolescents, especially those with psychotic disorders, exhibit more pronounced schizotypal personality disorder traits and report higher levels of emotional abuse compared to adults. This research underscores the importance of understanding age-related differences in psychological profiles to inform tailored clinical interventions and enhance treatment outcomes for diverse populations.

Taken together, the high-quality contributions gathered in this Research Topic provide valuable insights into the understanding and management of PD in youth, emphasizing the importance of early diagnosis and targeted treatment strategies. The articles collectively highlight the distinct characteristics of PD in younger populations, the impact of childhood experiences on their development, and the necessity for age-appropriate assessment tools. They advocate for a proactive approach in clinical settings, focusing on early intervention to mitigate the long-term effects of PD. Relatedly, this Research Topic serves to broaden understanding of the importance and imperative of providing effective early intervention, which hinges on providers’ abilities and confidence in diagnosing these disorders in youth. Furthermore, the discussions underscore the need for tailored therapeutic modalities that consider the unique developmental and psychosocial contexts of youth, thereby enhancing treatment efficacy and promoting healthier outcomes.

